# Evaluation of Ultraviolet Protection Claims Made by Umbrella Brands

**DOI:** 10.7759/cureus.71023

**Published:** 2024-10-07

**Authors:** Nia Gyongyosi, Grace E Axelson, Amelia Hilbert, Erum N Ilyas

**Affiliations:** 1 Dermatology, Drexel University College of Medicine, Philadelphia, USA; 2 Research and Development, UVtec, King of Prussia, USA

**Keywords:** beach umbrella, dermatology, patio umbrella, sun protection, sun protective measures, ultraviolet, ultraviolet protection factor (upf), upf umbrella

## Abstract

Introduction: The objective of this study was to review and evaluate umbrellas sold in the United States for sun protection. Seeking shade is among the current recommendations for protection against ultraviolet (UV) radiation, often through the use of handheld umbrellas (HU), beach umbrellas (BU), and patio umbrellas (PU). The study aims to assess the validity of UV protection factor (UPF) claims made by manufacturers and to determine if there is sufficient testing data to support these claims.

Methods: To evaluate consumer online shopping behavior for umbrellas for use as shade in the sun, keywords and keyword phrases were identified followed by Google (Google LLC, Mountain View, California, United States) and Amazon (Amazon.com, Inc., Seattle, Washington, United States) searches to determine brands and products retrieved. The top 13 brands and corresponding products were evaluated for their UV protection claims and validation of these claims.

Results: Of the 37 products including HU, BU, and PU, it was found that umbrellas for sun protection are more likely to be marketed with a UPF claim despite a 95% CI for a validated UPF claim of 2-10% with the breakdown for HU as (0%, 50%), BU (14.3%, 87.5%), and PU (0%, 0%). All UPF claims that were validated were only based on the new unused product and not after being subjected to wear and tear.

Conclusions: The marketing of umbrellas for use in the sun as a shade structure favors the use of UPF claims despite the absence of a standardized definition. The lack of validation for these claims along with inherent limitations of UV protection offered by shade structures suggests the UPF claim may be more of a promotional tactic than a reliable indicator of the UV protection provided. This underscores the need to continue recommending other sun-protective behaviors, such as wearing sunscreen and sun-protective clothing, even while using umbrellas.

## Introduction

Seeking shade is one of the most common recommendations for sun-safe behaviors with the use of umbrellas as high as 45% in Asia [1-3]. Of adults seeking sun-protective clothing, 76% prefer clothing products labeled with an ultraviolet protection factor (UPF) implying that the sun-savvy consumer may seek this labeling for verification of product claims in spite of the lack of consistency in determining UPF values assigned to shade structures [4-7].

This study aims to evaluate marketing practices that make use of claims for a UPF value by umbrellas targeting shade seeking consumers in the United States to determine the use and validation of these claims.

## Materials and methods

This study was conducted at the Drexel University College of Medicine, Philadelphia, Pennsylvania, United States. Online shopping behavior for umbrellas for use as shade in the sun (as opposed to rain) was evaluated by reviewing content from keyword and keyword phrase search terms. Umbrellas marketed for use in the sun are available in various forms such as handheld umbrellas (HU), beach umbrellas (BU), and patio umbrellas (PU). The top products were evaluated for their UV protection claims and validation of these claims. Simple statistics were used to analyze the data. This study, utilizing solely Internet search engines without the use of human subjects, was determined to be exempted from institutional review board approval.

Identification and selection of keywords

A total of 17 keywords and keyword phrases were identified for sun protective umbrellas based on popularity of use in search engines, as determined by search volume across three keyword tracking tools (Wordtracker [8], Ubersuggest [9], Google Keyword Planner [10]) beginning June 25, 2024 through July 5, 2024. Keyword-tracking tools monitor data and metrics to analyze user behavior to track and identify popular search terms and content preferences to support user needs. Each tracking tool uses different proprietary strategies to track and evaluate data. The three tools used in this study were utilized to validate search volume as a measure of popularity for each of the search phrases chosen. The search terms were selected to prioritize scenarios where users are specifically seeking umbrellas designed for use in the sun as opposed to other weather-related purposes. These terms were “patio umbrella”, “beach umbrella”, “outdoor sun umbrella”, “outdoor umbrella”, “pool umbrella”, “sun umbrella”, “UV umbrella”, “patio sun umbrella”, “UPF umbrella”, “beach UV umbrella”, “sun umbrella handheld”, “handheld umbrella”, “handheld UV umbrella”, “handheld UPF umbrella”, “patio UPF umbrella”, “patio UV umbrella”, and “beach UPF umbrella”. The 17 terms were entered into each keyword search tool separately, the volume of results recorded, then ranked from highest to lowest set for the United States, the English language, and the past 12 months. The top 10 keyword phrases based on the volume of searches were selected for this study. 

Identification and selection of brands

The content for each keyword phrase related to online shopping was analyzed by entering the phrases into Google (Google LLC, Mountain View, California, United States) and Amazon (Amazon.com, Inc., Seattle, Washington, United States) search engines between July 6 and July 9, 2024. Personalized search settings were disabled to ensure unbiased results. The top 10 search results for each keyword phrase from both Google and Amazon were recorded.

The “COUNTIF”, “ARRAYFORMULA”, and “REGEXMATCH” functions were used to determine how many times each brand appeared in the search results and the brands were arranged in descending order from highest to lowest number of occurrences. The 13 brands with the highest number of occurrences were selected for evaluation in our study.

Evaluation of brands

The top 13 brands were listed along with corresponding products for each. For each product, the product name, model number, use indication (PU, BU, HU), UPF rating claimed, materials used, additional sun protection features, and validation of UPF claim were recorded based on the availability of testing data. The availability of testing data to support each product's UPF claim was initially checked on the brand's product page on Amazon as well as the brand's official website. Additionally, companies were contacted via email to request this data. Each company was given a one-week timeframe from the day of contact to respond. Any UPF data either obtained from the brand’s website or sent from the brand itself was examined and recorded. The UPF data provided was assessed to verify whether the UPF claims used in marketing were supported by actual testing and to identify the specific UPF testing protocols used to confirm the claimed values. A claim was considered validated if any support for UPF testing was provided regardless of the protocol used. 

## Results

**Figure 1:**
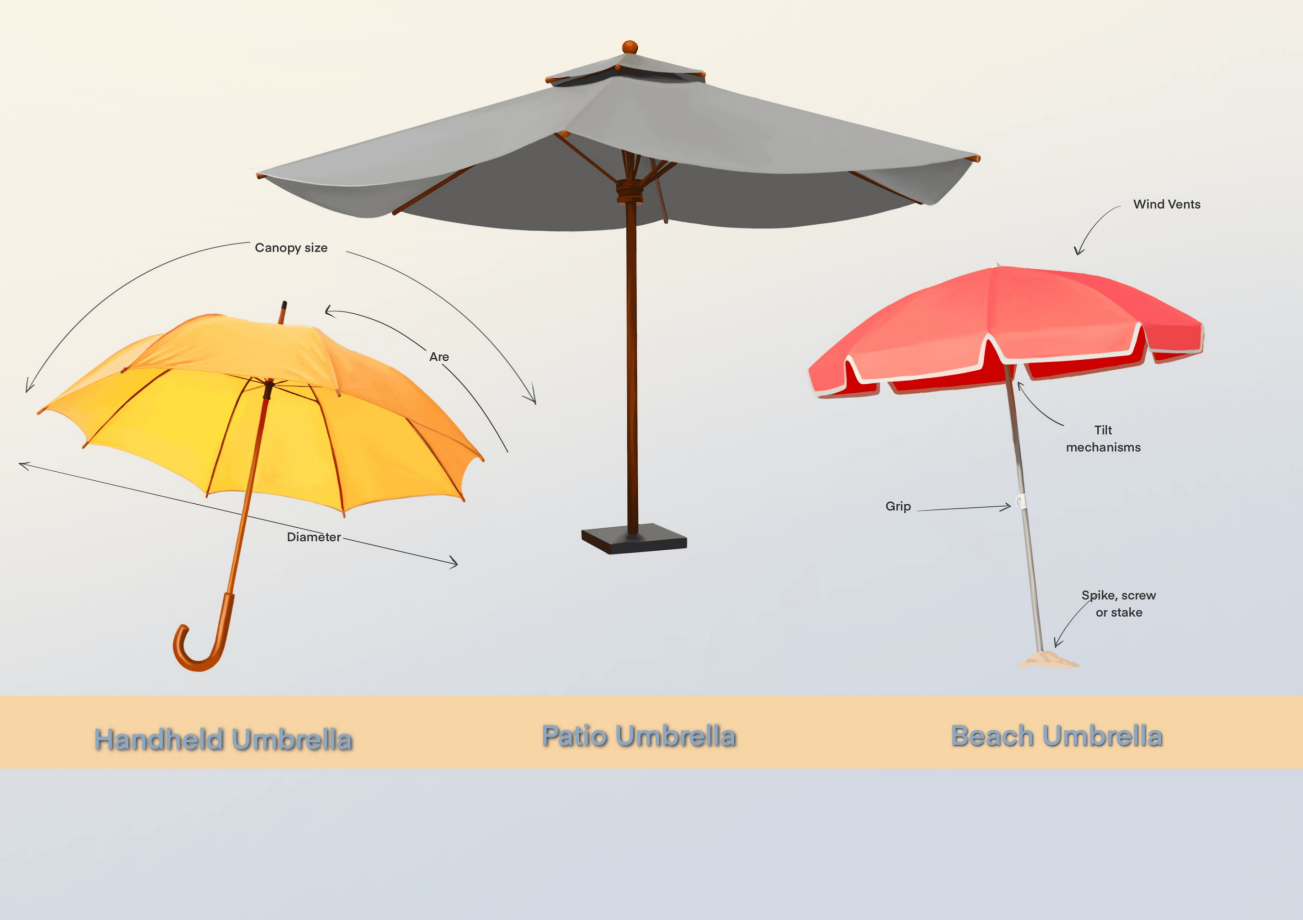
Forms and characteristics of umbrellas marketed for sun protection Image Credit: Artist Serena L; copyright owned by Erum Ilyas, MD

There were 17 keyword phrases with the volume of searches tracked and ranked based on popularity with the top 10 keyword phrases chosen to evaluate (Table 1). A total of 71 brands were found, with the top 13 chosen based on the volume of occurrences and 37 products (10 HU, 8 BU, and 19 PU) were evaluated (Figure 1).

**Table 1:** Total number of occurrences of keyword phrases for umbrella searches for use in UV ranked based on volume *Wordtracker [8] volume is based on average search volume per month over the past 12 months. **Ubersuggest [9] volume is based on the number of searches a keyword has in a particular month. ***Google Keyword Planner [10] volume is based on the average number of times people search for a keyword per month. UV: ultraviolet; UPF: ultraviolet protection factor

Search Terms	Wordtracker (Frequency of occurence)	Ubersuggest (Frequency of occurence)	Google Keyword Planner (Frequency of occurence)
Patio Umbrella	8300	90,500	10-100K
Beach Umbrella	7900	74,000	10-100K
Outdoor Sun Umbrella	44	33,100	10-100K
Outdoor Umbrella	5900	33,100	10-100K
Pool Umbrella	1200	12,100	10-100K
Sun Umbrella	2600	8100	1-10K
UV Umbrella	750	3600	1-10K
Patio Sun Umbrella	90	1300	<1K
UPF Umbrella	135	390	100-1K
Beach UV Umbrella	180	140	<1K
Sun Umbrella Handheld	28	140	<1K
Handheld Umbrella	44	90	100-1K
Handheld UV Umbrella	28	0	<1K
Handheld UPF Umbrella	n/a	0	<1K
Patio UPF Umbrella	12	0	<1K
Patio UV Umbrella	110	0	<1K
Beach UPF Umbrella	28	0	<1K

HU results

Of the 10 HU products evaluated, all made claims to UPF 50+ (100%) with two offering a UPF of 55+. Two (20%) of these products offered a UPF report on request with testing protocol AATCC 183 (American Association of Textile Chemists and Colorists Method 183) utilized to support UPF claims and offered claims of approvals or recommendations from outside sources such as “dermatologist recommended” and “Melanoma International Foundation” while 80% did not provide support for UPF testing data upon request nor was it available on their websites. Based on this data, the probability of finding an HU marketed with a UPF value is 100% while finding one with a validated UPF claim is approximately 20%. 

BU results

Of the eight BU evaluated, seven (87.5%) made claims for UPF 50+. Four of these products were made by the same brand, and offered a partial report from a third-party testing agency confirming a UPF >50; however, this report did not provide data beyond UVA and UVB blockage by the textile at a point in time nor details of the testing protocol. Considering the partial report as validation of UPF claims, the probability of finding a BU with a validated UPF claim is approximately 57%. 

PU results

Of 19 PU products evaluated, five stated a UPF value: four a UPF of 50+, and one an inconsistent claim of both 30+ and 50+ in different portions of the product page. Six offered claims of “UV resistant”, two with “sun resistant”, two with “UV protection”, and one with “UV protected”. One brand responded to a customer’s inquiry in the Q&A section of their product page stating that the branded fabric they use “blocks 97% of harmful sun rays”, but otherwise made no clarification as to which rays are blocked nor testing protocol used (visible light, UV, etc.). Two made no claims about UPF nor other vague claims about sun or UV protection or resistance. Only three of the 19 patio umbrella products responded to inquiries of UPF claims stating that testing was performed but not available. Based on this data, there is an 84% probability of finding a PU marketed with UV and sun references, 31% of which reference a UPF, and none with a validated claim. 

Statistical analyses

Evaluating the distribution of UPF, the CIs were calculated using a binomial proportion CI to provide a range in which the true proportion of validated UPF claims would likely fall based on the data observed (Table 2). For umbrellas overall there is a 95% CI for a validated UPF claim of 2% to 10% with the breakdown for HU as (0%, 50%), BU (14.3%, 87.5%), and PU (0%, 0%).

**Table 2:** Probability of finding an umbrella with UV features for HU, BU, and PU, validation of claims, and confidence intervals for finding a validated UPF claim. *UV references includes claims for "UV Resistant", "UV Protection", "UV Protected", "Sun Resistant", and "... will serve to protect your skin from the sun’s radiation..." while excluding UPF claims. UV: ultraviolet; HU: handheld; BU: beach umbrella; PU: patio umbrella; UPF: ultraviolet protection factor

Umbrella Type	Total Umbrellas	UPF Claims	Validated UPF Claims	UV References*	Proportion with UV References*	Proportion of UPF Claims	Proportion of Validated Claims	Exact 95% CI for Validated Claims
Handheld	10	10	2	0	0.00%	100%	20%	(0%, 50%)
Beach	8	7	4	0	0.00%	87.50%	57.10%	(14.30%, 85.70%)
Patio	19	5	0	12	63.16%	26.30%	0%	(0%, 0%)
Overall	37	22	6	12	32.40%	59.50%	27.30%	(2%, 10%)

A chi-square test was performed to determine if there is a statistically significant difference in the distribution of search engine results for UPF claims, UV references, and no UV references across the umbrella categories (Table 3). With 59.5% of products (n=22) making UPF claims, 32.4% (n=12) with UV references, and 8.1% (n=3) with no UV references, the chi-square statistic of 19.84 (p-value = 0.00054) indicates a significant difference in the distribution of search engine results. This result suggests that products referencing UPF values are favored by search engines than those making vague references to UV or not referencing UV at all. A p-value of less than 0.001 was considered significant for this test.

**Table 3:** Chi-Square test results for the association of UPF Claims, UV references, and no UV references and search engine results for umbrellas for use in the sun UV: ultraviolet; UPF: ultraviolet protection factor

Test	Chi-Square Value	Degrees of Freedom	p-Value	Effect Size (Cramer's V)
UPF Claims vs. UV References vs. No UV References	19.84	4	0.00054	0.52

## Discussion

The American Academy of Dermatology (AAD) recommends shade as an effective sun-protective practice making use of an umbrella infographic without specific reference to the use of umbrellas as a shading structure [11]. It should be noted that the overall construction of the shade structure determines the exposure levels to UV directly underneath. It is essential to consider the entire shading structure, not just the material, as it should have enough overhang to prevent high levels of scattered radiation from reaching the skin [5]. The ability of the fabric used in the construction of the umbrella to consistently offer sustained UV protection can be dictated by the weave, color, composition, and durability through wear and tear; however, the protection is only appreciable in the areas directly blocked by the textile [12]. The ability of umbrellas to block UV varies based on the material used with black nylon showing a range from 64.5% to 92.3% UV blockage and additional variability based on angle and location [3,13,14].

Given the variability in UV protection offered by textiles, a UPF claim has the potential to offer added confidence in the material’s ability to block UV. The UPF value is assigned to a product to indicate the ability of the material to directly block UVA and UVB radiation; however, this would not take into consideration angles and position underneath the structure [3,5,6]. Additionally, testing methods to determine UPF may not replicate real-life situations to account for wear over time such as with exposure of these materials to wind, rain, and fading. Different UPF testing protocols are intended to account for some of these differences in textiles used for outdoor shade structures. 

There is not a consistent standard applied to determining UPF values to assign to shade structures. Some testing protocols test textiles only in the new and unused state such as AATCC 183, while others may subject the textile to wear and tear to offer a higher degree of validation to a sustained UPF claim through standard product use such as the UV Standard 801. AS/NZS 4399:1996 and AS/NZS 4399:2017, used in Australia/New Zealand, and EN13758, used in Europe, are both comparable to AATCC 183 as they test textiles only in the new and unused state [15-17]. This is not preferable as UPF often drastically decreases as fabric wears, stretches, and abrades with use and environmental exposure [16]. ASTM 6544 (American Society for Testing and Materials 6544) employs the same measurement methodology as described above but subjects test samples to factors such as laundering, light exposure, chlorinated pool water, and perspiration [15]. These various testing conditions more accurately reflect real-life conditions that can affect the amount of UV transmittance or blockage. Similarly, UV Standard 801 takes into account the influence of usage conditions and evaluates the lowest UV protection offered by textiles in wet, stretched, and artificially weathered conditions [15,16]. For accuracy purposes, choosing testing protocols that best resemble the practical use of the textile is of great importance for consumer protection and to validate claims. 

In evaluating umbrella products in this study, although 100% of HU, 87.5% of BU, and 26.32% of PU made UPF claims, only 20% of HU and 57% of BU while none of the PU were able to offer some proof of UPF testing and all were in the new and unused state. Search engines are favoring products that reference UPF values over those that make vague references to UV or do not reference UV at all in spite of the lack of validated UPF claims.

Consumers shopping online make use of keywords and keyword phrases to describe intent in performing a search. These keywords are tracked and algorithms associated with search engines search for content on web pages to provide results consistent with assumed user preferences. The results of this study demonstrate that search engines tend to favor products that reference UPF with a p-value <0.001. This data underscores the importance of validated UPF testing to provide consumers with confidence in the UV protection sought. Although this study accepted any proof of UPF testing as validation of claim, consumers would benefit from knowing whether the UPF rating was based on new and unused materials and/or artificially weathered materials. 

Of note, the greatest variety in UV references and claims was seen with PU. Out of 19 PU, 26.32% offered UPF claims that could not be validated while 63.16% of the remainder made vague UV references. Common phrases used included “UV resistant”, “sun resistant”, “UV protected”, “UV protection”, and lastly "... blocks 97% of harmful sun rays...". It should be noted that the phrase “UV resistant” is generally used to reference the impact of UV on color fading of the textile used and not UV blockage offered by the shade structure. UV absorbers may be added to these textiles upon dyeing and finishing to absorb UV and reduce fading of the product [18]. Reference to UV resistance may impact search engine results in online shopping but may not offer consumers a product that has been tested specifically for blocking UV penetration through the fabric.

One brand utilized medical support and claimed their product to be “Approved by Melanoma International Foundation”, “Dermatologist Tested”, and “Dermatologist Recommended”. Verification of approval by the Melanoma International Foundation, and further detail into the meaning behind the claim “Dermatologist Tested” could not be verified on the product’s web page. However, this was also the only brand that offered verified and clear UPF testing data upon request with documentation supporting the AATCC 183 protocol that tests textiles for UV blockage in the new, unused state and not after exposure to wear and tear or other environmental factors.

Limitations

There are notable limitations to the study. By making use of keywords and phrases through online search tools, our data is limited to the online shopping experience and assumptions made to phrases to be searched based on search volume tracked. This study does not consider the in-store shopping experience for the average consumer seeking sun-protective products without product “vetting” by a search engine. The results of this study focused on the volume of searches and products returned by searches to maximize understanding of the most likely products to be searched and found. Furthermore, while personal bias was limited with personalization settings turned off in used web browsers, there is no way to validate that all search results were void of previous browser history influences. A similar study could be performed by tracking products available in stores; however, challenges may persist based on inventory available and geography served by individual retail. This study is also limited by the availability of data provided by manufacturers regarding their UPF claims. This evaluation is based solely on the information and materials accessible at the time of the study. Additionally, given the vast number of brands with umbrellas sold online, cataloging every brand offering UV-protective umbrellas was not reasonable. The focus on the highest volume was intended to capture the most likely results. However, individuals may shop through social media and influencer marketing, which is not necessarily captured by these searches. Although the volume may have been captured by these searches, there are brands that cater to the sun protective needs of their consumers that may comply with more stringent criteria for UV protection for their products. However, only one of the products evaluated claimed recommendation by a health resource implying that brands that may cater to specifically sun protective needs are either not favored by search engines by keyword optimization strategies or handle a smaller volume of consumers. Nonetheless, our search results as based on volume demonstrated that there is not a high volume of consumers seeking out or discovering smaller brands. 

## Conclusions

This study highlights that the marketing of umbrellas for use in the sun as a shade structure favors the use of UPF claims despite the absence of a standardized definition, which can lead to consumer confusion. The lack of validation for these claims along with inherent limitations of UV protection offered by shade structures suggests the UPF claim may be more of a promotional tactic than a reliable indicator of the UV protection provided. This underscores the need to continue recommending other sun-protective behaviors such as wearing sunscreen and sun-protective clothing, even while using umbrellas.
